# Gas chromatography for analysis and estimation of ^13^C at natural abundance level in fatty acids produced from *Aurantiochytrium limacinum*, a sustainable source of polyunsaturated fatty acid

**DOI:** 10.3389/fbioe.2025.1631063

**Published:** 2025-07-22

**Authors:** Amina M. Dirir, Kaumeel Chokshi, Abdelmoneim H. Ali, Media Alhanawi, Mohan Rommala, Mayssa Hachem

**Affiliations:** ^1^ Department of Chemistry, College of Engineering and Physical Sciences, Khalifa University of Sciences and Technology, Abu Dhabi, United Arab Emirates; ^2^ Food Security and Technology Center, Khalifa University of Sciences and Technology, Abu Dhabi, United Arab Emirates; ^3^ Department of Food Science, College of Agriculture and Veterinary Medicine, United Arab Emirates University (UAEU), Al Ain, United Arab Emirates; ^4^ Analytical Chemistry and Biochemistry Core Laboratories, Khalifa University of Sciences and Technology, Abu Dhabi, United Arab Emirates

**Keywords:** *Aurantiochytrium limacinum*, PUFA, DHA, CSIA, GC-FID, GC-C-IRMS

## Abstract

*Aurantiochytrium limacinum (A. limacinum)* is a promising microbial source of polyunsaturated fatty acids (PUFAs), particularly Docosahexaenoic Acid (DHA, C22:6n-3). In this study, we first optimized the culture conditions of *A. limacinum* ATCC MYA-1381 (strain SR21). Cell growth was monitored *via* optical density, cell counts, and glucose concentration. Cells were harvested at exponential and stationary phases, and lipids were extracted using a green method. Fatty Acid Methyl Esters (FAMEs) were prepared and analyzed using Gas Chromatography-Flame Ionisation Detection (GC-FID). At the exponential phase, DHA was the most abundant (65.6% of total fatty acids) followed by palmitic acid (C16:0) at 34.4%. At the stationary phase, Docosapentaenoic acid (DPA, C22:5n-3) and DHA were the most abundant at 45.4% and 33.9%, before respectively. Myristic acid (C14:0), myristoleic acid (C14:1n-9), palmitic acid (C16:0) were present at 4.6%, 6.2% and 9.9%, respectively. Compound-specific isotope analysis (CSIA) using Gas Chromatography-Combustion-Isotope Ratio Mass Spectrometry (GC-C-IRMS) revealed that all FAMEs had negative δ^13^C values, indicating depletion in ^13^C. At the exponential phase, δ^13^C (‰) of C16:0 and DHA were −16.8 ± 0.2 and −18.5‰ ± 0.1‰, respectively. At the stationary phase, δ^13^C (‰) of C14:0, C14:1n-9, C16:0, C22:5n-3 and DHA were −10.6 ± 1.1, −11.3 ± 0.1, −11.1 ± 0.2, −8.3 ± 0.2 and −10.6‰ ± 0.1‰, respectively. Overall, our findings emphasized the importance of *A. limacinum* as a viable microbial platform for environmentally friendly production of PUFA such as DHA. Also, the study reinforced the utility of CSIA in tracking PUFA metabolic fate, which has latent applications in biomedical research, particularly in neurodegenerative disease frameworks where DHA plays a vital role. Finally, these results may also contribute to understanding isotopic fractionation patterns and metabolic flux variations across different microalgal growth phases.

## 1 Introduction

Docosahexaenoic acid (DHA, C22:6n-3) is an omega-3 polyunsaturated fatty acid (PUFA) mainly found in human’s brain and eyes exhibiting several beneficial health effects mainly in prevention and potential treatment of neurodegenerative diseases such as Alzheimer’s and Parkinson’s in addition to eyes’ diseases (Hachem and Nacir, 2022; Hachem et al., 2016; Belkouch et al., 2016; Hachem et al., 2020). DHA can be found in several food sources including fish and salmon. Moreover, DHA can be produced from microalgae.

Indeed, algae are a group of photosynthetic organisms found in various aquatic ecosystems, such as rivers, oceans, and ponds. They can tolerate the variation of several environmental parameters, including temperature, salinity, pH values, and light intensity. They can also endure harsh conditions in deserts, reservoirs and grow alone or in symbiosis with other organisms (Khan et al., 2018). Moreover, algae encompass various groups of photosynthetic organisms including microscopic unicellular microalgae and complex macroalgae, also known as seaweed. More recently, algae have received substantial attention due to several features, including remarkable nutritional profiles and potentials in food and pharmaceutical applications (Wu et al., 2023; Kumar et al., 2021; Kent et al., 2015).

Similar to other photosynthetic organisms, algae can synthesize various bioactive compounds known as secondary metabolites (Cardozo et al., 2007; Pereira et al., 2021). Metabolites are produced at the end of the growth phase or due to abiotic/biotic stress induced by the surrounding environmental conditions. These bioactive natural metabolites produced from algae including phycobiliprotein pigments, phenolic compounds, carotenoids and polysaccharides exhibit a wide range of biological properties, such as anticancer, antimicrobial, and antioxidant activities (Shalaby, 2011; Roleira et al., 2015; Avila-Roman et al., 2021; Hou and Cui, 2018). Additionally, microalgae can produce several lipids-based compounds, including fatty acids (FAs) and PUFAs such as DHA (Remize et al., 2021). FAs are considered as one of the major groups of microalgae biomass and generally constitute 5%–60% of cell dry weight (Morales et al., 2021).

Among those microalgae, Thraustochytrids, categorized as Stramenopiles, are heterotrophic marine protists capable of producing vast quantities of PUFA including DHA (Nyunoya et al., 2022). Additionally, *Crypthecodinium cohnii* is another heterotrophic marine dinoflagellate considered as a rich producer of DHA. DHA’s purification process from *C. Cohnii* is appealing since it significantly produces DHA, rather than any other PUFAs, utilized in pharmaceutical and nutraceutical applications (Mendes et al., 2009). Moreover, *Isochrysis galbana* is an example of microalgae producing DHA. Indeed, *I. galbana* can grow in harsh environmental conditions and possess a high growth rate and productivity. It is considered also as a rich source for other valuable products significant for human health such as vitamins, polysaccharides, sterols and carotenoids (Señoráns et al., 2020). Likewise, *Nannochloropsis oculata* is one more rich source of DHA and EPA (Nuño et al., 2013). Furthermore, *Aurantiochytrium limacinum (A. Limacinum)*, a thraustochytrid strain, is considered an ideal source of DHA constituting 1.43%–29.6% of total FAs (Sirirak et al., 2020) where the composition of FAs is generally determined as fatty acids methyl esters (FAMEs) using Gas Chromatography coupled with Flame Ionisation Detection (GC-FID) (Ichihara and Fukubayashi, 2010).

While GC-FID approach was extensively applied for FAs profiling in microalgae (Visudtiphole et al., 2021; Dillon et al., 2019; Jarle Horn and Klüver, 2025), the combination of the GC power with the sensitivity of Isotope Ratio Mass Spectrometry (IRMS) was recently introduced to microalgal systems, through analysis of stable isotope ratios (carbon-13, hydrogen-2, etc.) of individual molecules. This approach is known as Compound-Specific Isotope Analysis (CSIA) (Pilecky et al., 2021; Xin et al., 2024). In CSIA, the isotopic composition is expressed as δ^13^C values, which denote the ratio of ^13^C–^12^C in a sample relative to a standard (Mašalaitė et al., 2012). Indeed, δ^13^C-based metabolic tracing is a prevailing approach used to investigate lipids’ biosynthesis in microalgae through following the stream of ^13^C-labeled carbon in metabolic pathways (Twining et al., 2020; Remize et al., 2022). Through analysis of δ^13^C values of diverse lipids, researchers can identify the active metabolic pathways in lipid biosynthesis (Zhang and Liu, 2021) and how fast carbon flows *via* these pathways (Xin et al., 2024; Meier-Augenstein, 2002; Rhee et al., 1997).

Despite the application of CSIA in microalgal systems, no fundamental research was conducted till day on the application of Gas Chromatography-Combustion-Isotope Ratio Mass Spectrometry (GC-C-IRMS) for CSIA at natural abundance levels mainly in relation to DHA biosynthesis and metabolic flux changes between growth phases in *A. limacinum*. Hence, the objective of the present study was to first optimize the culture of *A. limacinum* and investigate the FAs profiling through GC-FID. Then, we developed a novel CSIA approach to estimate δ^13^C values at natural abundance level in FAs produced using GC-C-IRMS.

## 2 Materials

### 2.1 Chemicals

Solvents including hexane, methanol, and chloroform were purchased from Honeywell (USA). Sodium chloride, glucose, yeast extract, and boron trifluoride (methanol solution, 14% in methanol) were obtained from Sigma-Aldrich (USA). Peptone was brought from HiMedia (India), and the antibiotics, including ampicillin, kanamycin monosulfate salt, and streptomycin sulfate, were obtained from Glentham (UK). Isooctane and sulfuric acid were purchased from Supelco (USA), toluene from Fisher Chemical (USA), and potassium carbonate from ACS Reagents (USA). Vitamin E was obtained from Ambeed (USA).

### 2.2 Instrumentation

An orbital shaker from Kethink (China) was used for cell cultivation, and cell counting was performed using a microscope from Celestron (USA). Optical density measurements were performed using a UV-Visible spectrophotometer from ThermoFischer Scientific (USA). Glucose concentration was quantified using a High-Performance Liquid Chromatography (HPLC) system from Waters Alliance 2,695 (USA). For biomass and lipids collection, a centrifuge from Electrical (China), a 4.5 L Cascade Benchtop freeze-drying system from Labconco (USA), a Reacti-Therm heating and stirring model from ThermoFischer Scientific (USA), a microwave from Black and Decker (USA) and a digital ultrasonic cleaner from Daihan (South Korea) were used. Gas Chromatography with Flame Ionization Detector (GC-FID) from Agilent Technologies 7890B GC System (USA) and Gas Chromatography-Combustion-Isotope Ratio Mass Spectrometry (GC-C-IRMS) from ThermoFischer Scientific (Germany) were used for FAs profiling and CSIA, respectively.

## 3 Methods

### 3.1 Culture of *Aurantiochytrium limacinum*

*Aurantiochytrium limacinum (A. limacinum)* ATCC MYA-1381 cells, designated as strain SR21 and deposited as *Schizochytrium limacinum* Honda et Yokochi, were obtained from American Type Culture Collection (ATCC). The cells were grown in ATCC-790 medium containing 5 g glucose, 1 g peptone and 1 g yeast extract in 1 L of filtered seawater collected from the Persian Gulf in Abu Dhabi, UAE. The culture medium was supplied with 100 mg/L of ampicillin, kanamycin and streptomycin. 275 μL of the cryopreserved *A. limacinum* ATCC MYA-1381 cells were added into 500 mL flasks containing 200 mL of the culture medium. Cultures were incubated at 150 rpm in a shaker maintained at 25°C until the cells reached an exponential and stationary phase. All experiments were carried out in triplicates.

### 3.2 Growth parameters

2 mL of culture was collected daily for measuring three different parameters: (i) optical density of the cells, (ii) cell count and (iii) glucose concentration in the culture medium.

#### 3.2.1 Optical density of the cells

Cell growth was monitored daily through measurement of the optical density. 1 mL of the culture was collected from each flask and the optical density was measured at 660 nm (OD_660_) using a UV-Vis spectrophotometer (Chodchoey and Verduyn, 2012).

#### 3.2.2 Cell count

Cell counting was performed using a hemocytometer and a Celestron LCD Digital Microscope. Briefly, 90 µL of the culture was mixed with 10 µL of ethanol. 6 μL of the mixture was loaded onto the hemocytometer and the number of cells were counted manually. The final cell count was calculated using Equation 1.

$$\text{Cell number }\left(\text{cells}/\text{ml}\right)=\text{Average cell counts from each square}\times 10^{4}$$ (1)

#### 3.2.3 Glucose dosage in culture medium through high performance liquid chromatography

To monitor glucose consumption and determine the exponential and stationary phases, glucose levels in the culture medium were measured using HPLC. 1mL of the culture medium was filtered using a 0.22 µm filter and the filtrate was used to estimate the residual glucose concentration using an Aminex HPX-87H Column (300 × 7.8 mm, Bio-Rad, USA) as a stationary phase and 5 mM H_2_SO_4_ as a mobile phase. The column temperature was maintained at 30°C and the flow rate was set 0.6 mL per minute. On day 0, 1 mL of the culture medium was collected to measure the initial glucose concentration. A calibration curve was prepared using glucose standards in the range of 0.5–6 g/L (*R* ^2^ = 0.98).

### 3.3 Cell harvesting

Based on glucose measurements, once glucose became limiting, we identified the exponential and stationary phases and interrupted the cultures to collect the biomass followed by extraction of lipids. Cultures were harvested by centrifugation at 450 *g* for 10 min at 4°C. The cell pellets were collected, lyophilized, weighed and stored at −20°C for further analysis.

### 3.4 Lipid extraction

Lyophilized biomass was used for the extraction of lipids using microwave-assisted extraction (MAE), a green extraction technique. For MAE approach, 9% sodium chloride (NaCl) was added to the biomass to get 5% (w/v) solid following standard method (Zghaibi et al., 2019). 500 W of power (low-medium setting) was applied in short pulses (20 s ‘on’ followed by 20 s of immediate ice cooling) over a total of 5 min to prevent thermal degradation of lipids. This pulsed mode of heating was chosen to maintain lipid integrity while ensuring efficient cell wall disruption. Following the extraction, 5 mL of hexane was added to recover the extracted lipids into non-polar organic phase. The mixture was centrifuged at 450 *g* for 5 min and the organic phase was collected and evaporated under nitrogen stream. The dried samples were stored at −20°C.

### 3.5 FAME production

To determine the FA composition of lipids extracted from *A. limacinum*, we produced the methyl esters of fatty acids (FAME) for GC-FID analysis (Iverson and Sheppard, 1965). The saponification followed by methylation is a conventional technique for preparation of FAMEs (Ackman, 1998). FAMEs were produced according to a modified AOAC 969.33 method with toluene/methanol (2:3, v/v) and BF_3_/methanol (14%) where BF3 is a catalyst for methylation and methanolysis (Ackman, 1998). The mixture was heated at 100°C for 90 min. The reaction was stopped by immersing the tubes in ice and 10% of potassium carbonate (K_2_CO_3_) and isooctane were added. Following, the mixture was centrifuged at 450 *g*, 5 min, 4°C and the organic phase containing FAMEs was recovered. The solvent was evaporated under nitrogen and FAMEs were stored at −20°C until analysis.

### 3.6 Gas chromatography-flame ionisation detection (GC-FID) analysis

For FAs profiling and quantification, we conducted a GC-FID analysis. The system consists of an Agilent 7890B GC-FID with Rt-2560 GC Capillary Column (100 m × 0.25 mm x 0.20 µm) purchased from Restek. The carrier gas was helium at a constant flow of 2 mL/min. The FID was set at 280°C, with a gas flow of 350, 35, and 30 mL/min of synthetic air, hydrogen, and helium, respectively. The injected sample volume was 1 μL. A series of blank (Hexane, HPLC grade) and 37 FAME standard mix procured from Restek were prepared in GC vials and measured in parallel to the samples to qualitatively identify and quantify each FA in the total FAs extracted per sample. Initially, the column temperature was set at 80°C and held for 10 min. Following, the temperature was increased at the rate of 7 °C/min until it reached 170°C, where it was maintained for 10 min. Another temperature increase was set at 12 °C/min to 205°C, followed by a 20 min hold and 20 °C/min until reaching 220°C, with a hold for 15 min. Lastly, the temperature was increased at 15 °C/min to 230°C and maintained for 20 min.

### 3.7 Gas chromatography isotope ratio mass spectrometry (GC-IRMS) analysis

#### 3.7.1 ^13^C isotopic analysis of FAMEs

Compound Specific Isotopic Analysis CSIA of prepared FAMEs from *A. limacinum* was conducted using a Gas Chromatography-Combustion-Isotope Ratio Mass Spectrometry GC-C-IRMS, DELTA™ Q Isotope Ratio Mass Spectrometer from Thermo Fischer Scientific. FAMEs (1 µL) were injected in a spitless operating mode with a TriPlus RSH autosampler (Thermo Scientific, Bremen, Germany) onto a SP^®^-2560 Capillary GC Column L × I.D. 100 m × 0.25 mm (Supelco) fixed in a Trace 1310 GC (Thermo Fischer Scientific). In order to reach a suitable signal, all samples were prepared at 100 ng of FAMEs per 1 µL of hexane. A standard of 37 Food Industry FAME Mix, procured from Restek was analysed applying the same protocol followed for all samples, allowing to identify individually each FAME.

Complete baseline separation of diverse FA from surrounding peaks was accomplished by the subsequent temperature ramp: initial temperature of 100°C held for 4 min, increasing to 200 °C at 25°C/min and held for 8 min, then to 250°C at 5°C/min and held for 6 min. The total run per sample was 33 min. Separate FAMEs eluting off the column were directed by He carrier gas to a GC IsoLink II interface functioning at 1,000°C with a Ni, CuO and Pt combustion reactor set at 1,000°C (Thermo Fisher Scientific, Bremen, Germany). FAMEs entering the combustion reactor were quantitatively combusted to CO_2_ and water. Water formed was trapped by a Nafion^®^ dryer (DuPont, Wilmington, DE) and only CO_2_ was introduced into the Delta V Plus IRMS (Thermo Scientific) *via* a ConFlo IV Universal Interface (Thermo Scientific) with continuous flow interface. Electron ionization voltage was 77 eV, electron current was 1.5 mA, and 3 F cup collectors for m/z 44, 45, and 46 were used for detection of CO_2_.

#### 3.7.2 Isotopic normalization

**FIGURE 1:**
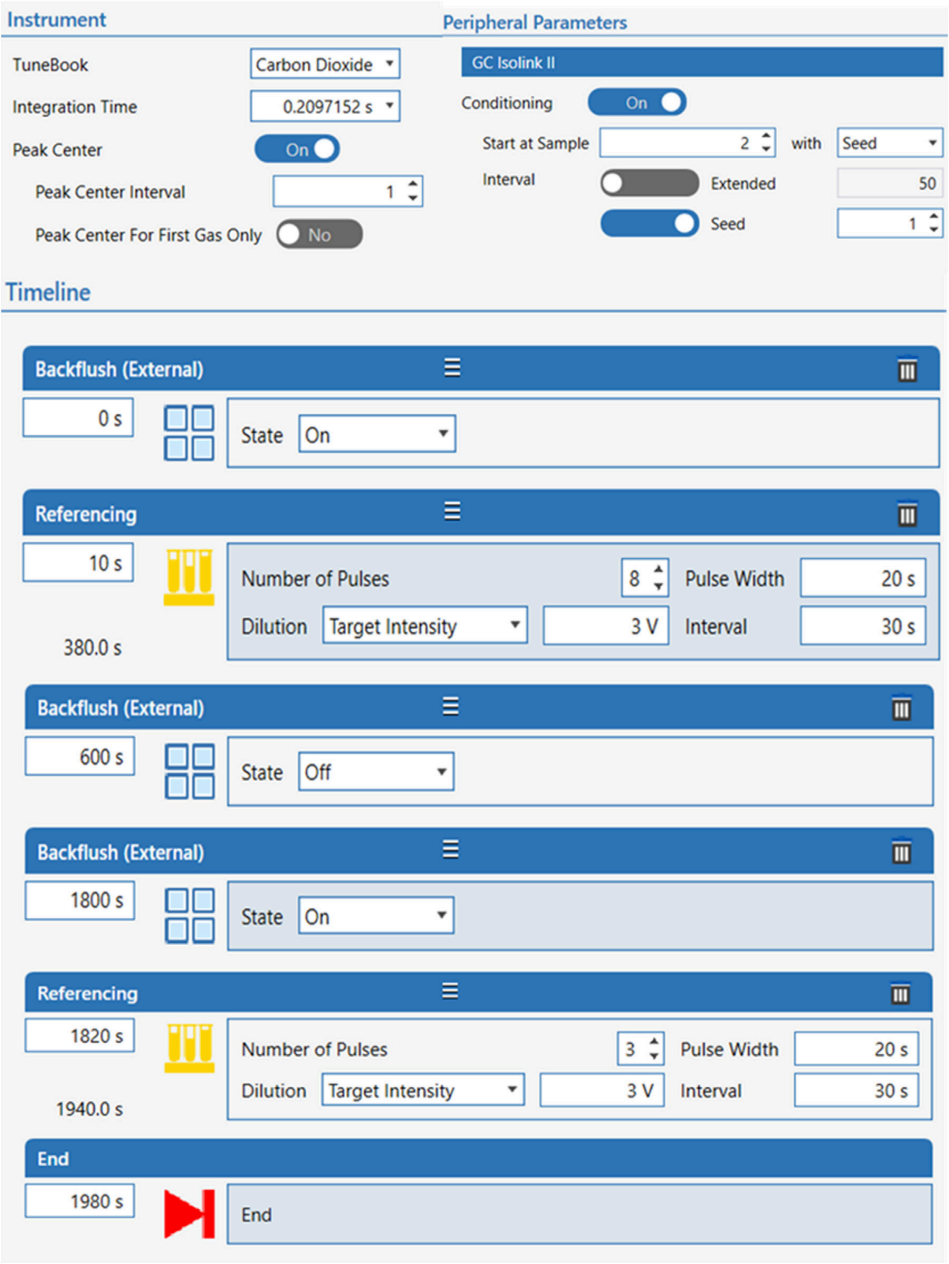
Conditions of CO_2_ continuous flow for isotope ratios calibration.

**FIGURE 2:**
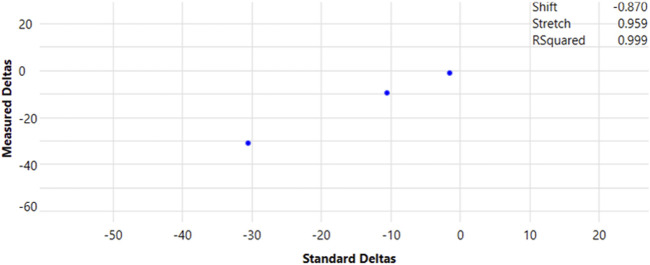
Multipoint normalization curve for the reporting of true δ^13^C values for GC-IRMS data. FAME (20-carbon) certified reference materials, USGS70, USGS71, and USGS72, were injected periodically during each programmed sequence, totalling at least three injections per run.

Carbon isotopic data collected by IRMS were normalized and described as per mil (‰; 1‰ = 0.001 = 1 millurey or mUr). Isotope ratios were calibrated against reference CO_2_ of known isotopic composition introduced directly into the ion source eight times at the beginning and three times at the end of every run following below conditions for continuous flow CO_2_ (Figure 1). Raw δ^13^C isotopic values obtained relative to the CO_2_ working gas from GC-IRMS analysis, were converted to the international carbon isotope reference scale, Vienna Peedee Belemnite (VPDB), by multi-point linear normalization (Coplen et al., 2006). USGS certified FAME reference materials (USGS70, USGS71, and USGS72) were injected every five samples throughout each analytical sequence. Each standard was injected at least three times per sequence, including at the beginning, midpoint, and end of the run. Multi-point linear normalization was applied based on the known δ^13^C values of the standards, with *R* ^2^ values exceeding 0.999, ensuring high analytical precision. Linear regression of measured values *versus* true one (−30.5‰ ± 0.04‰, −10.5‰ ± 0.03‰, and −1.5‰ ± 0.03‰ for USGS70, USGS71, and USGS72, respectively) generated a normalizing equation, which was subsequently applied to report true δ^13^C values for all data (Figure 2).

## 4 Results

### 4.1 Growth parameters

#### 4.1.1 Optical density of cells

**FIGURE 3:**
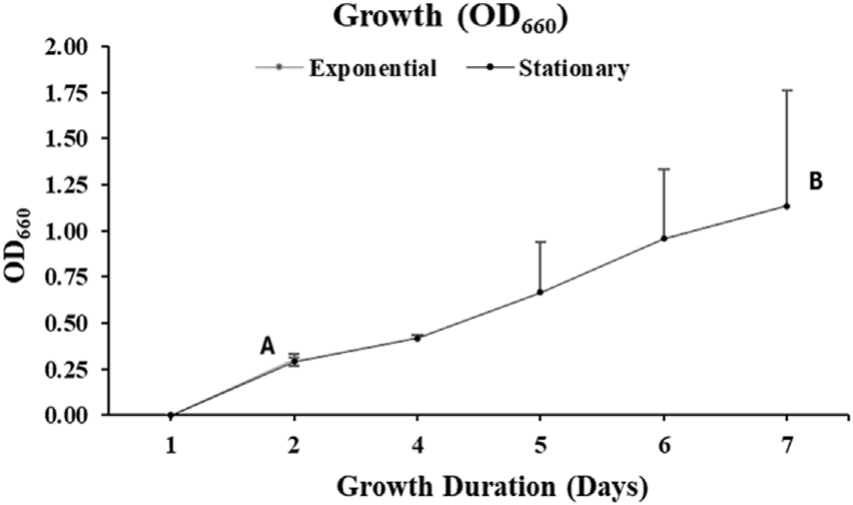
Cellular absorbance measured by UV-Visible Spectrophotometry at wavelength of 660 nm of the culture of *Aurantiochytrium limacinum* ATCC MYA-1381 in 200 mL of medium under control condition ^12^C-glucose. (A) Exponential phase culture (B) Stationary phase culture. Values are expressed as means+/-SD (n = 3).

*Aurantiochytrium limacinum* ATCC MYA-1381 cell growth was monitored daily by measuring OD_660_. As illustrated in Figure 3, cells harvested on day 2, at exponential phase reported an OD_660_ of 0.3 ± 0.03. At stationary phase, on day 7, OD_660_ of the cells was 1.1 ± 0.6.

#### 4.1.2 Cells counts

**FIGURE 4:**
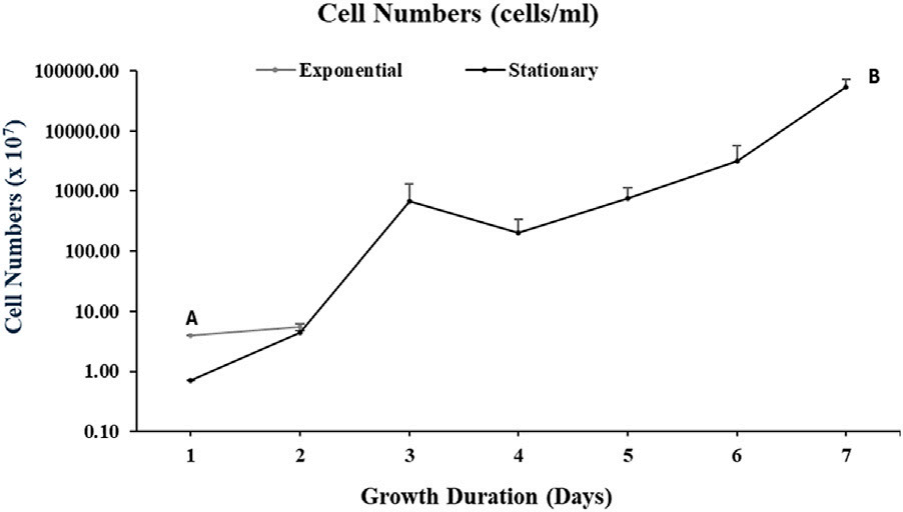
Cell counts of *Aurantiochytrium limacinum* ATCC MYA-1381 in 200 mL of medium under control condition ^12^C-glucose. (A) Exponential phase culture (B) Stationary phase culture. Values are expressed as means+/-SD (n = 3)

As illustrated in Figure 4, for the cultures harvested at an exponential phase, the cell number was 5.53 × 10^6^ per ml of the culture whereas at stationary phase, the cell number was 5.35 × 10^10^ per ml of culture.

#### 4.1.3 Glucose dosage in culture medium using high performance liquid chromatography

**FIGURE 5:**
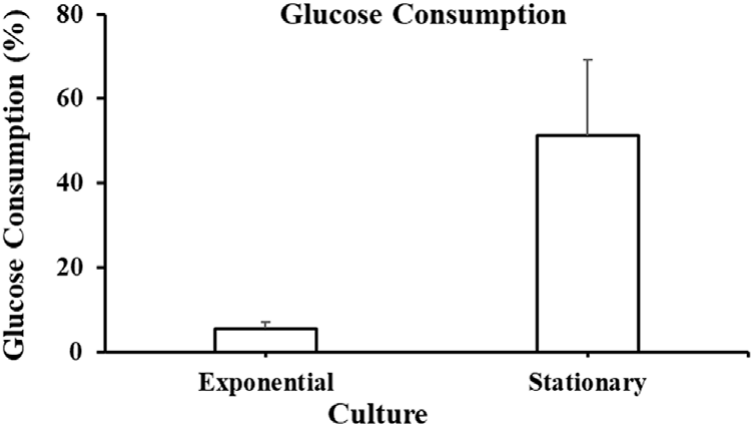
Glucose consumption at exponential and stationary phase cultures of *Aurantiochytrium limacinum* ATCC MYA-1381 on the day of harvesting. Values are expressed as means+/-SD (n = 3).

Figure 5 shows glucose consumed by *A. limacinum* ATCC MYA-1381 cells on harvesting day (Day 2 for exponential and Day 7 for stationary phase). The cells harvested at the exponential phase consumed 5.5% ± 1.5% of glucose, while those harvested at the stationary phase consumed 51% ± 18% of glucose.

### 4.2 Biomass collection and lipids extraction

**FIGURE 6:**
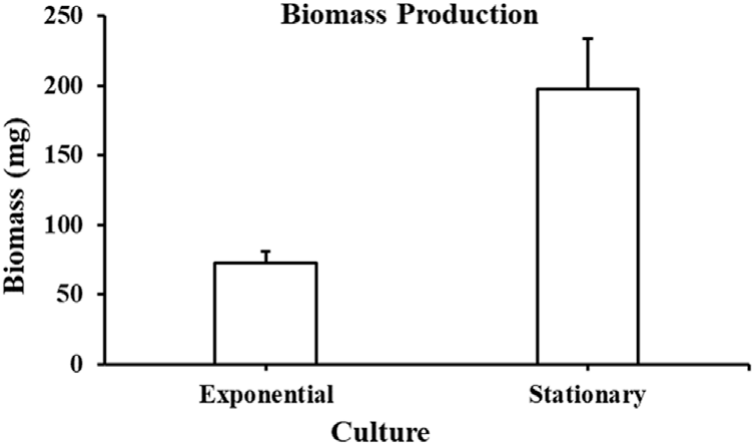
Biomass collected from *Aurantiochytrium limacinum* ATCC MYA-1381 at exponential and stationary phase cultures. Values are expressed as means+/-SD (n = 3).

Biomass produced from *A. limacinum* ATCC MYA-1381 cells is represented in Figure 6. At exponential phase, the biomass collected was 72 ± 9 mg whereas at stationary phase, the biomass production was 197 ± 37 mg after 7 days of cultivation. Total lipids at exponential and stationary phases were 11.8 mg and 102 mg, respectively.

### 4.3 GC-FID analysis

**FIGURE 7:**
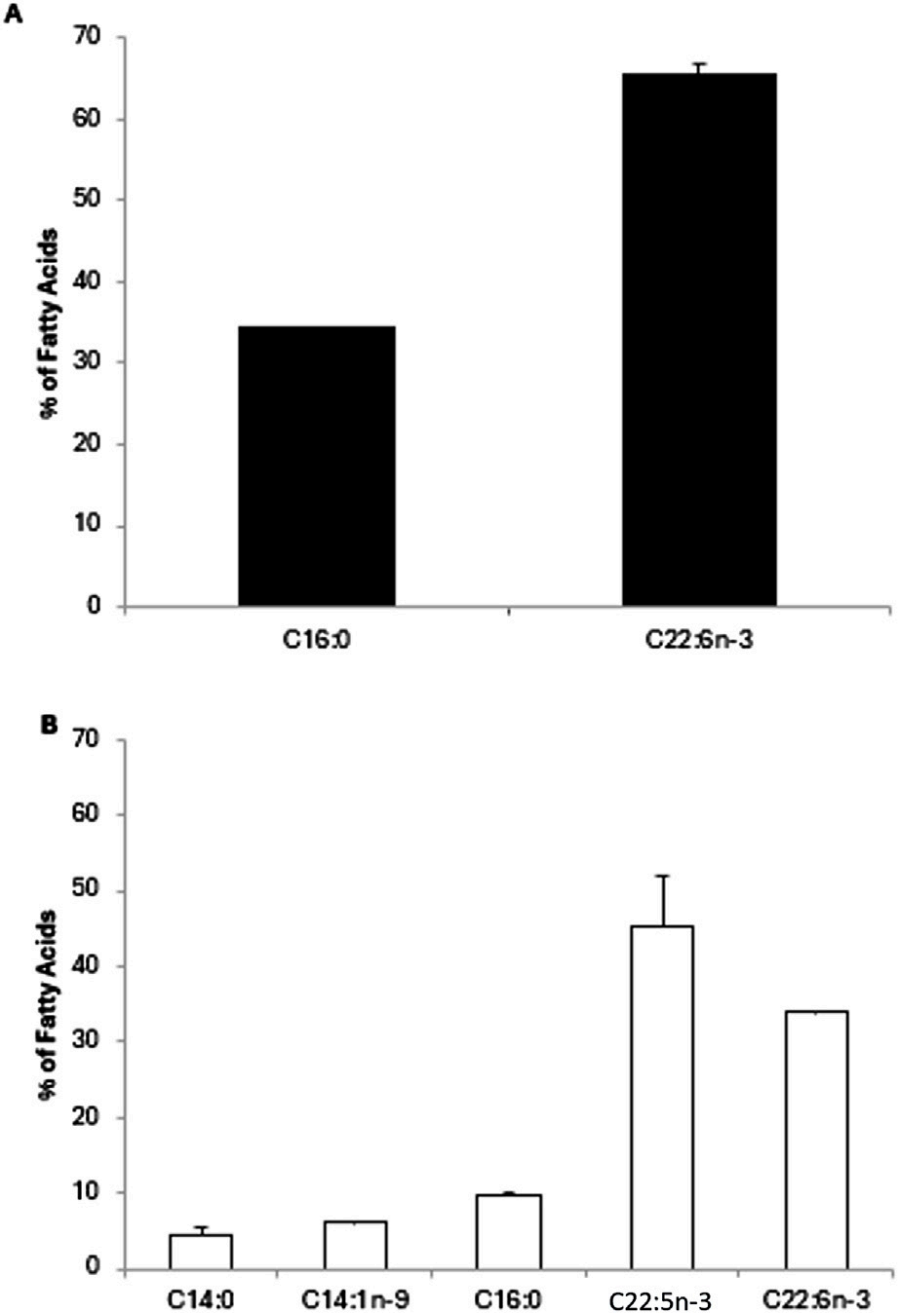
Fatty acid composition of *Aurantiochytrium limacinum* ATCC MYA-1381 in 200 mL of culture medium under control conditions at exponential **(A)** and stationary phase **(B)** using GC-FID analysis. Results are expressed as percentage of FA and expressed as means+/-SD (n = 3).

Following total lipid extraction and FAMEs production, we performed a GC-FID analysis and calculated the percentage of individual FAs. For *A. limacinum* cultures harvested at an exponential phase (Figure 7A), two major FAs were identified. DHA was the most abundant with 65.5% ± 1.1% of total FAs followed by palmitic acid (C16:0) with 34.4% ± 0.1%. At stationary phase (Figure 7B), Docosapentaenoic acid (DPA, C22:5n-3) and DHA were the most abundant FAs with 45% ± 7% and 33.9% ± 0.1%, respectively. Additionally, myristic acid (C14:0), myristoleic acid (C14:1n-9), C16:0 were observed at 4.6% ± 0.8%, 6.2% ± 0.1% and 9.8% ± 0.1%, respectively.

### 4.4 GC-IRMS analysis

#### 4.4.1 Isotopic signatures of reference materials USGS70, USGS71 and USGS72

IRMS is a powerful technique to quantify variations in the isotopic composition of several elements including carbon. An approach that has proven successful is to measure the ^13^C-enrichment level. Differences in ^13^C-enrichment level are expressed using the delta notation shown below in Equation 2:

$$\delta^{13}C\frac{0}{00}=\frac{R_{sample}}{R_{standard}}-1\times 1000$$ (2)

where R is the ratio of the number of atoms of the minor isotope to that of the major isotope (Brand and Coplen, 2012).

**FIGURE 8:**
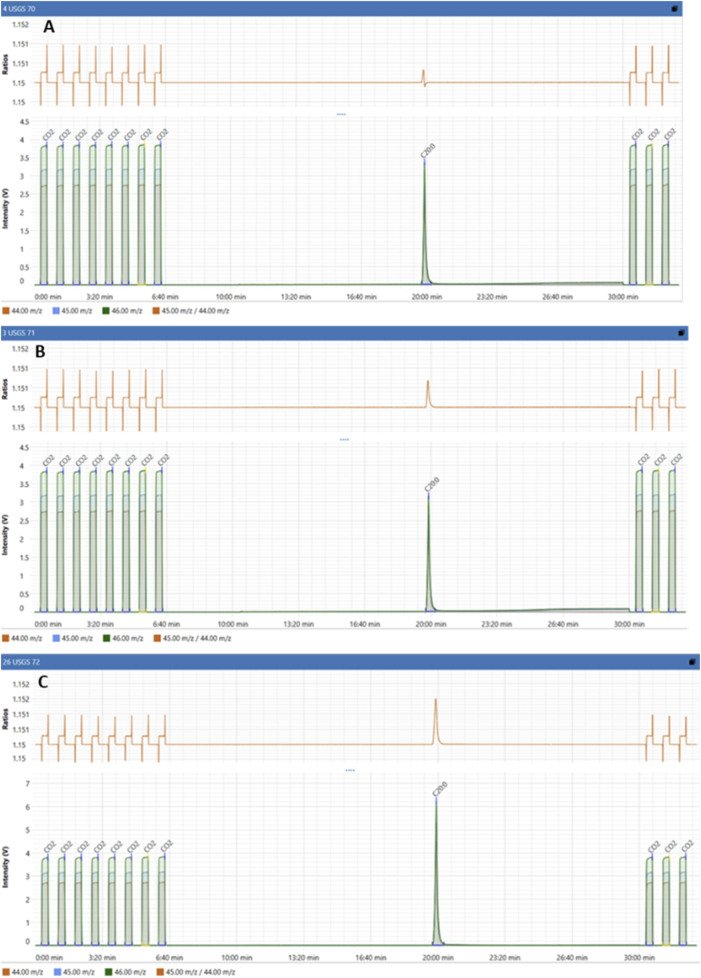
Isotopic signatures of Reference Materials: **(A)** USGS70, **(B)** USGS71 and **(C)** USGS72.

To compare isotope enrichment levels, a standard reference material (RM) is used. For all RM (USGS70, USGS71, USGS72), in addition to the chromatogram showing the intensity (V) *versus* retention time (min), we plotted the isotopic swing S shape *versus* time (min) (Figure 8). Since all RM had the same composition which is icosanoic acid methyl esters (C_20_H_39_O_2_CH_3_), we identified their retention time of 19.5 min. Furthermore, we noticed excellent profiles of isotopic swing as well as good resolution for CO_2_ measurements.

#### 4.4.2 Isotopic signatures of FAME produced from *Aurantiochytrium limacinum* at exponential and stationary phase

**FIGURE 9:**
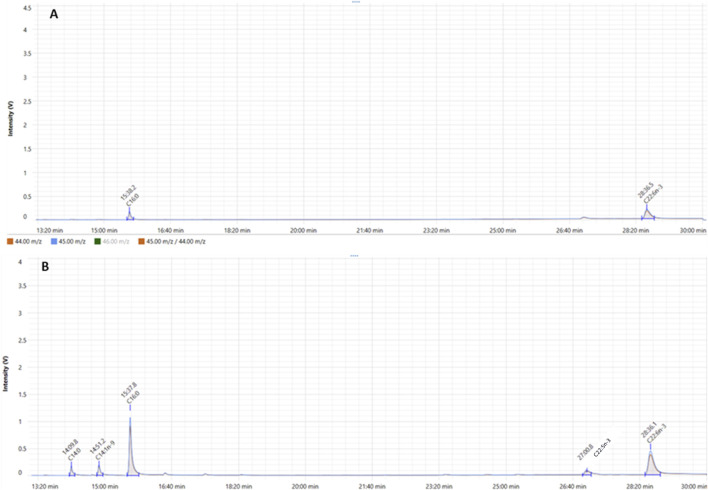
GC-IRMS analysis of FAMEs derived from total lipid extracted from *Aurantiochytrium limacinum* at exponential phase **(A)** and stationary phase **(B)**. The orange plot shows the signal ratio for m/z 45/44. The blue plot shows the signal for m/z 45 for the duration of the IRMS run.

**FIGURE 10:**
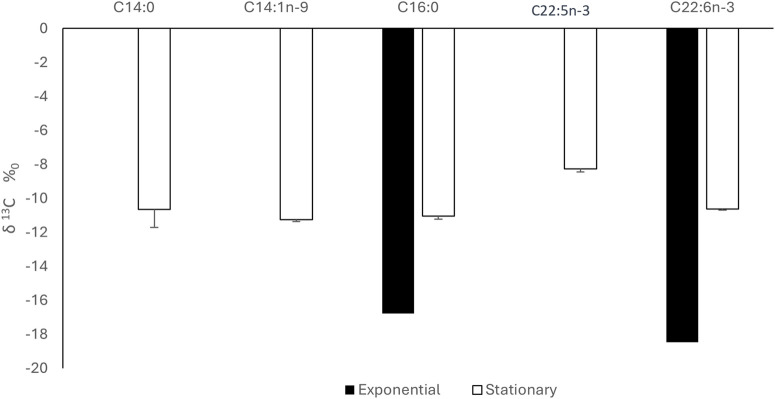
δ^13^C signature of FAME at exponential and stationary phase using GC-C-IRMS analysis. Results are expressed as percentage of FA and expressed as means+/-SD (n = 3).

As previously explained, CSIA of FAMEs produced from microalgae was conducted using GC-C-IRMS where we measured the isotopic C13 signature of each FA, expressed as δ^13^C (‰). At exponential phase, C16:0 and DHA were identified in comparison to standards with retention time of 15.3 and 28.4 min, respectively (Figure 9A). At stationary phase, in addition to C16:0 and DHA, three FAs were eluted at 14.1, 14.5 and 27 min which were identified as C14:0, C14:1n-9 and C22:5n-3, respectively (Figure 9B). δ^13^C signature of each FAME was quantified and results show that all FAMEs produced at different growth stages had negative δ^13^C values (Figure 10). This means that FAMEs were depleted in ^13^C, which is normal since the cells were cultivated in the presence of ^12^C-glucose and not ^13^C-glucose. As shown in Figure 10, δ^13^C (‰) of C16:0 and DHA at exponential phase were −16.8 ± 0.2 and −18.5‰ ± 0.1‰, respectively, At stationary phase, δ^13^C (‰) of C14:0, C14:1n-9, C16:0, C22:5n-3 and DHA were −10.6 ± 1.1, −11.3 ± 0.1, −11.1 ± 0.2, −8.3 ± 0.2 and −10.6‰ ± 0.1‰. respectively.

## 5 Discussion

The life cycles of thraustochytrids are extremely reliant on nutrition. When cultivated in artificial media comprising yeast extract or peptone, thraustochytrids can accumulate great quantities of biomass and lipids, making them very attractive for biotechnological applications (Dellero et al., 2018). It is well recognised that lipid production is influenced by the composition of the culture media and differs with the life cycle. Such fluctuations are associated with significant environmental/cultivation restrictions including the C:N ratio. Certainly, a carbon source is mandatory for these heterotrophic cells to produce FAs and lipids. This carbon source can be assumed through glucose or glycerol addition to the culture media. The FA compositions of several thraustochytrid types have been investigated in order to evaluate their content in terms of several PUFAs mainly DHA. Diverse culture environments (changing the source of carbon, oxygen concentration, salinity, temperature, nitrogen accessibility, etc.) were tested to determine the effect of the culture medium on FAs’ production and unsaturation (Zhu et al., 2007). Previous researchers have showed that thraustochytrid types produced mainly C16:0 and DHA, the latter varying from one species to another. The genera *Aurantiochytrium*, and *Schizochytrium* were the most productive of DHA (Fossier Marchan et al., 2018). Certainly, the medium composition is an important parameter, the presence of glucose or glycerol as a carbon source and yeast extract or peptone as a nitrogen source allowing the highest yield (Aasen et al., 2016).

In the present study, *A. limacinum* cells were grown on glucose with a natural carbon isotope abundance in the presence of peptone, yeast extract as a nitrogen source allowing the highest yield and sea water collected from Persian Gulf. To avoid bacterial proliferation, three different antibiotics were added to the culture medium. All experiments were conducted in biological triplicates. Within 2 days of cultivation, the cell count was 5.53 × 10^6^ per ml of the culture, whereas, after 7 days, the cell count increased by a factor of 10^4^ and reached 5.35 × 10^10^ per ml of the culture. No bacterial growth or contamination were observed during the full culture’s period.

Glucose decreased in the culture medium to 51% ± 18% on day 7. We could have increased the cultivation duration to 9 or 10 days, but it could be risky mainly related to bacterial proliferation and death of cells. The carbon conversion yield, defined as the amount of biomass produced per Gram of substrate consumed, was 0.08 g biomass/g glucose. This fairly low conversion yield is steady with literature values for *Aurantiochytrium* strains with comparable heterotrophic cultivation, where an important fraction of carbon is oriented toward lipid accumulation (especially PUFAs) rather than biomass. Moreover, some carbon may be missed as CO_2_ as a result of respiratory metabolism or diverted into non-biomass cellular components including extracellular polysaccharides or secondary metabolites. Furthermore, the high standard deviation observed in glucose consumption might represent natural biological variability among different cultures. During the stationary phase, this could reflect metabolic heterogeneity in response to nutrients in *A. limacinum* influencing glucose consumption and lipid biosynthesis.

A good quantity of biomass was collected with 72 ± 8 mg and 197 ± 36 mg on day 2 and day 7 of cultivation, respectively. Total lipids produced were around 16% and 52% on day 2 and day 7 of cultivation, respectively and these results aligned well with other researchers who reported that *A. limacinum* typically accumulates lipids up 30%–60% of its dry weight lipids at stationary phase (Hoshino et al., 2016). From the FAs profiling, we observed that at an exponential phase, DHA was the most copious FAs with 65.6% of total FAs followed by C16:0 with 34.4%. However, at the stationary phase, DPA and DHA were the most abundant FAs with 45.4% and 33.9%, respectively. Furthermore, C14:0, C14:1n-9, C16:0 were 4.6%, 6.2% and 9.9%, respectively. Indeed, the pragmatic decline in DHA abundance from 65.6% in the exponential phase to 33.9% in the stationary phase might be an indicative of a metabolic flux redirection, probably related to the physiological transition of *A. limacinum* from active growth to lipids’ accumulation. Throughout the exponential phase, cells prioritize membrane lipid biosynthesis and speedy proliferation (Soto-Sánchez et al., 2023), with high DHA level playing a fundamental structural role in PL-rich cellular membranes. Contrarily, during the stationary phase, the metabolic focus moves toward the accretion of storage lipids mainly triacylglycerols (TAGs), less enhanced in DHA and more connected to saturated and monounsaturated FAs. This metabolic rerouting might decrease the flux toward PUFA biosynthesis, counting DHA, thus dropping its proportion in total FAs pool. These findings are aligned with those in the literature showing that, when thraustochytrids including *Aurantiochytrium* and *Schizochytrium* are grown up in suitable environments, they can accumulate DHA with almost around half of the total FAs (Coplen et al., 2006; Brand and Coplen, 2012).

When investigating the CSIA using GC-C-IRMS at ^13^C natural abundance level in FAs, we found that δ^13^C signature of all FAME were negative suggesting that FAME were depleted in ^13^C since the cells were grown on ^12^C-glucose and not ^13^C. Indeed, for negative values of δ^13^C, the carbon isotope fractionation often happens in the process of fatty acids biosynthesis, and more ^12^C is used. Thus, providing a lower δ^13^C (Kelleway et al., 2018). The δ^13^C (‰) of C16:0 and DHA were −16.8 ± 0.2 and −18.5‰ ± 0.1‰, respectively, at the exponential phase while at the stationary phase, δ^13^C (‰) of C14:0, C14:1n-9, C16:0, C22:5n-3 and DHA were −10.6 ± 1.1, −11.3 ± 0.1, −11.1 ± 0.2, −8.3 ± 0.2 and −10.6‰ ± 0.1‰. respectively.

Notably, the exponential phase displays more negative δ^13^C values for DHA at −18.5‰ compared to the isotopic enrichment of DHA in the stationary phase with −10.6‰, reflecting a further direct and effective accommodation of ^12^C-rich glucose *via* central carbon metabolism and suggesting a shift in carbon source utilization or carbon allocation efficiency between growth phases. As glucose concentration decrease in the culture medium, cells might use internal carbon reserves or alternatives, which have a tendency to be comparatively ^13^C-enriched. Moreover, the recycling of intracellular carbon *via* anaplerotic paths or the improved involvement of endogenous CO_2_ fixation may decrease isotope fractionation, ensuing less negative δ^13^C values (Bowling et al., 2008). Together, these results support the hypothesis that carbon flux redirection and nutrient-driven metabolic remodeling meaningfully impact both FAs profiles and their isotopic signatures throughout diverse growth phases (Kobayashi et al., 2020; Mang Gao, 2013; D.H. et al., 1998).

During the stationary phase, the δ^13^C value of DPA was remarkably less negative (−8.3‰) compared to DHA (−10.6‰). This isotopic enrichment in DPA may reflect underlying variances in metabolic pathways and enzyme activity during FA biosynthesis. Additionally, DPA and DHA biosynthesis in *A. limacinum* likely proceeds *via* the polyketide synthase (PKS)-like pathway or the elongase/desaturase pathway. In the latter case, DHA is naturally derived from DPA *via* a Δ4 desaturase enzyme, and DPA in turn is synthesized from eicosatetraenoic acid (ETA) or arachidonic acid through elongation and Δ6 desaturation. The less negative δ^13^C of DPA may consequently indicate that it serves as an intermediate pool which accumulates during the stationary phase, possibly due to reduced Δ4 desaturase activity or altered enzyme kinetics under nutrient-depleted conditions. Furthermore, this isotopic pattern might be linked to carbon source allocation preferences and fractionation effects. The carbon flux during the stationary phase is likely redirected toward energy storage and structural lipid synthesis, with selective incorporation of isotopically heavier carbon (^13^C-enriched) into certain intermediates like DPA. This could occur due to kinetic isotope effects in elongase or desaturase enzymes or due to precursor channeling dynamics where isotopically lighter carbon is preferentially routed to DHA synthesis or other metabolic sinks.

Furthermore, at stationary phase, the higher standard deviation detected for the δ^13^C value of C14:0 (±1.1‰) is steady with its lower relative abundance and better sensitivity to slight shifts in precursor accessibility or enzymatic turnover. Since C14:0 is a trivial FA in the stationary phase, minor variations in its biosynthesis might lead to amplified isotopic variance. Thus, these differential δ^13^C signatures provide insight into the dynamic regulation of PUFA biosynthesis under varying physiological conditions and supports the utility of GC-C-IRMS in elucidating pathway-level isotope fractionation.

The newly developed CSIA method can be applied in the context of several studies focusing on targeting the brain with PUFAs, especially DHA to estimate the ^13^C natural abundance level in FAs. To purify different FAs including DHA produced from *A. limacinum*, several approaches could be applied including solid phase extraction (SPE) as well as HPLC. The purified PUFAs including DPA and DHA can have several beneficial applications mainly in nutrition and health sectors.

## 6 Conclusion

*Aurantiochytrium limacinum* can be considered as a sustainable and biotechnological source for DHA production for medical applications. The newly developed CSIA at natural abundance levels in microalgal systems could allow a better understanding of the dynamic regulation of PUFA biosynthesis as well as the metabolic flux discrepancies between growth phases supporting the significance of GC-C-IRMS in explicating pathway-level isotope fractionation.

## Data Availability

The original contributions presented in the study are included in the article/supplementary material, further inquiries can be directed to the corresponding author.
